# Role of Microvessel Density and Vascular Endothelial Growth Factor in Angiogenesis of Hematological Malignancies

**DOI:** 10.1155/2016/5043483

**Published:** 2016-02-22

**Authors:** Rashika Chand, Harish Chandra, Smita Chandra, Sanjiv Kumar Verma

**Affiliations:** ^1^Department of Pathology, Himalayan Institute of Medical Sciences, Swami Ram Nagar, Doiwala, Dehradun, Uttarakhand 248140, India; ^2^Department of Medicine, Himalayan Institute of Medical Sciences, Swami Ram Nagar, Doiwala, Dehradun, Uttarakhand 248140, India

## Abstract

Angiogenesis plays an important role in progression of tumor with vascular endothelial growth factor (VEGF) being key proangiogenic factor. It was intended to study angiogenesis in different hematological malignancies by quantifying expression of VEGF and MVD in bone marrow biopsy along with serum VEGF levels and observing its change following therapy. The study included 50 cases of hematological malignancies which were followed for one month after initial therapy along with 30 controls. All of them were subjected to immunostaining by anti-VEGF and factor VIII antibodies on bone marrow biopsy along with the measurement of serum VEGF levels. Significantly higher pretreatment VEGF scores, serum VEGF levels, and MVD were observed in cases as compared to controls (*p* < 0.05). The highest VEGF score and serum VEGF were observed in chronic myeloid leukemia and maximum MVD in Non-Hodgkin's Lymphoma. Significant decrease in serum VEGF levels after treatment was observed in all hematological malignancies except for AML. To conclude angiogenesis plays an important role in pathogenesis of all the hematological malignancies as reflected by increased VEGF expression and MVD in bone marrow biopsy along with increased serum VEGF level. The decrease in serum VEGF level after therapy further supports this view and also lays the importance of anti angiogenic therapy.

## 1. Introduction

Angiogenesis plays an important role in progression of tumor along with metastasis and invasion. It is a complex process which is mediated by angiogenic and antiangiogenic factors. Vascular endothelial growth factor (VEGF) and basic fibroblast growth factor (bFGF) are the key proangiogenic factors which interact with tyrosine kinase receptors and enhance endothelial cell proliferation and increased vascular permeability [1, 2]. Although the role of angiogenesis in solid tumors is well established, its importance in hematological malignancies is being studied widely especially in reference to different types of leukemia, their prognosis, and therapeutic implications [3, 4]. The quantification of angiogenesis may involve the measurement of microvessel density (MVD) and serum VEGF levels supported by the expression of VEGF in bone marrow biopsies. Different studies have given variable results regarding VEGF expression in various hematological malignancies with some showing increased expression while others concluding that no difference exists in VEGF expression between hematological malignancies and controls [2, 5]. The present study was therefore conducted to study angiogenesis in different types of hematological malignancies by studying the expression of VEGF and MVD in bone marrow biopsy along with measurement of serum VEGF levels. It was also intended to study effect of therapy on angiogenesis by observing the change in serum VEGF levels following initial/induction therapy.

## 2. Material and Methods

The study included 50 new cases of hematological malignancies which were diagnosed on bone marrow examination and followed up for at least one month after initial therapy. Thirty controls were included in the study which were newly diagnosed cases of lymphoma or solid tumor malignancies but without the evidence of bone marrow infiltration. All the cases and controls were subjected to immunostaining by anti-VEGF and factor VIII antibodies on bone marrow biopsy (Biogenex, California, USA) along with the measurement of serum VEGF level by using the principle of enzyme linked immunosorbent assay (ELISA) at the time of diagnosis before treatment. Serum VEGF level was also quantified in follow-up after at least one month after treatment. The immunohistochemical expression of VEGF was studied by giving the score of 0–3 according to staining intensity in immunopositive cells in at least 10 fields (×40, Olympus) and observed independently by two observers (score 0, no staining; score 1, mild staining; score 2, moderate staining; and score 3, severe staining). For quantitation of MVD, the hotspots on bone marrow biopsy (areas containing highest number of blood vessels) were identified by using immunohistochemical expression of factor VIII antibodies. This was followed by counting of total number of vessels (×100, Olympus) in at least five fields with each field representing an area of 0.392 m^2^. The true vessel number in the biopsy was then expressed as mean of five counts.

Statistical analysis was done using SPSS software version 17 and Student's *t*-test and correlation coefficient test were used to determine the statistical significance association. *p* < 0.05 was considered significant.

## 3. Results

**Figure 1:**
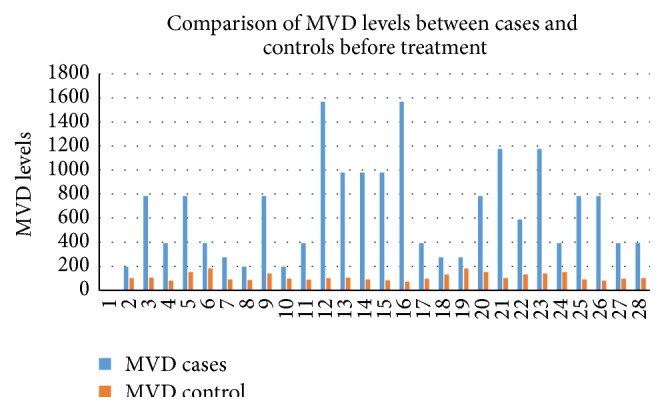
Column chart of MVD level pretreatment for cases and controls.

**Figure 2:**
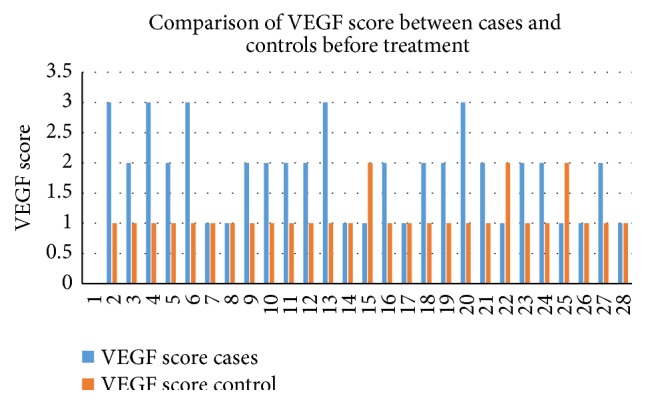
Column chart of VEGF expression pretreatment for cases and controls.

**Figure 3:**
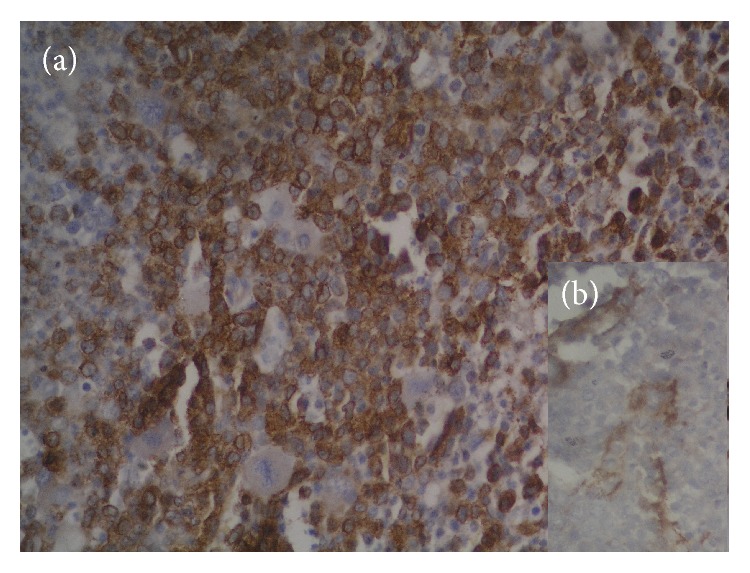
(a) Bone marrow biopsy showing VEGF immunoexpression with score 3 in CML (VEGF, ×400). (b) Bone marrow biopsy showing factor VIII immunoexpression for microvessel density in NHL (factor VIII, ×400).

The study included total 50 cases with male: female ratio of 1.5 : 1 and median age of 28.5 years with range of 5–75 years.  Table 1 shows the distribution of different hematological malignancy with mean age and sex ratio. It shows that acute myeloid leukemia (AML) and acute lymphoid leukemia (ALL) were the most common leukemia with each comprising 30% of total cases. All the malignancies showed preponderance of males except AML (male female ratio of 0.8 : 1). Figures 1 and 2 show significantly higher pretreatment VEGF scores and MVD in cases as compared to controls. The pretreatment serum VEGF levels were also significantly raised in comparison to controls (*p* < 0.05).  Table 2 shows average VEGF score and MVD in different hematological malignancy. All the hematological malignancies showed raised VEGF score and MVD with positive correlation coefficient (*r* = 0.1071). The highest VEGF score and serum VEGF level were observed in chronic myeloid leukemia (CML) and maximum MVD in Non-Hodgkin's Lymphoma (NHL) (Figure 3).  Table 3 shows significant decrease in serum VEGF levels after treatment in comparison to pretreatment levels in all the hematological malignancies except for AML. A positive correlation between serum VEGF level before treatment and after treatment (*r* = 0.6616) which was statistically significant (*p* < 0.0001) was observed.

## 4. Discussion

Sustained angiogenesis is considered to play an important role in progression and metastasis of tumors. Tumor angiogenesis is controlled by the balance between angiogenesis promoters and inhibitors and VEGF is an important prognostic cytokine [6, 7]. VEGF activates “Notch signaling pathways” to coordinate angiogenesis and upregulation of VEGF is influenced by mutations of RAS or MYC gene [7]. This has led to emergence of anti-VEGF drugs for treatment of cancers along with the strategies that inhibit “Notch Activation” [8]. VEGF along with microvessel density has been studied in different hematological malignancies for tumor angiogenesis. The present study observed that VEGF expression and MVD in bone marrow were increased in all the hematological malignancies including acute leukemias, chronic leukemias, multiple myeloma (MM), and NHL. It was observed that highest VEGF expression and serum VEGF levels were seen in CML indicating maximum angiogenic potential in it. Another important finding observed in the present study was significant decrease in serum VEGF level after treatment in follow-up further highlighting the importance of angiogenesis in pathogenesis of hematological malignancies. Further, a positive correlation coefficient was observed between VEGF score and MVD in all the hematological malignancies. Similar finding has also been observed by Gianelli et al. who concluded that VEGF expression correlates with MVD in Philadelphia negative chronic myeloproliferative disorders [2]. The highest MVD was seen in NHL followed by MM in the present study but El-Sorady et al. have observed that highest bone marrow microvessel count was present in MM suggesting higher angiogenic potential in such patients [9]. The observation of increased angiogenesis in all hematological malignancies in the present study especially CML and NHL indicates the potential use of anti-VEGF therapy therapies for their treatment. However studies have concluded that blocking of VEGF activity improves the delivery of cytotoxic drugs to tumor and endothelial cells but have also raised the question of importance of biomarkers to identify patients who may benefit from antiangiogenic treatment along with optimal dose and mechanism of resistance [10].

An important limitation of the present study was that angiogenesis was not studied in myelodysplastic syndrome (MDS) and its role in progression. However, previous studies have suggested that vascularity and angiogenic factors are increased in MDS and thus play an important role in leukemogenic process [11].

## 5. Conclusion

Thus to conclude angiogenesis plays an important role in pathogenesis of all the hematological malignancies including acute and chronic leukemia, lymphoma and multiple myeloma as reflected by increased VEGF expression, and MVD in bone marrow biopsy along with increased serum VEGF levels. The decrease in serum VEGF level after therapy further supports this view and also lays the importance of antiangiogenic therapy in all the hematological malignancies. In addition, further studies are needed to know the exact mechanism and interaction of VEGF with cellular components and bone marrow milieu which may affect prognosis, progression, and therapeutic outcome of these hematological malignancies.

## Figures and Tables

**Table 1:** Distribution of different types of hematological malignancy with mean age and sex ratio.

Type of malignancy	Total cases	Percentage of total cases (%)	Median age (years)	Male : female ratio
AML	15	30	35	0.8 : 1
ALL	15	30	14	2.7 : 1
CML	6	12	25.5	1 : 1
CLL	2	4	72.5	All males
MM	10	20	49	1 : 1
NHL	2	4	53	All males

AML, acute myeloid leukemia; ALL, acute lymphoid leukemia; CML, chronic myeloid leukemia; CLL, chronic lymphoid leukemia; MM, multiple myeloma; NHL, Non-Hodgkin's Lymphoma.

**Table 2:** Average VEGF score and MVD level in different hematological malignancy.

	VEGF score mean ± standard deviation	MVD level (microvessels/mm^2^) mean ± standard deviation
AML	2 ± 0.75	324 ± 173.09
ALL	1.26 ± 0.45	632.33 ± 413.57
CML	2.66 ± 0.51	555.33 ± 192.70
CLL	2 ± 0	333 ± 83.43
MM	2 ± 0.81	693.8 ± 266.61
NHL	2 ± 0	1372 ± 277.18

AML, acute myeloid leukemia; ALL, acute lymphoid leukemia; CML, chronic myeloid leukemia; CLL, chronic lymphoid leukemia; MM, multiple myeloma; NHL, Non-Hodgkin's Lymphoma.

**Table 3:** Pre- and posttreatment serum VEGF levels in different hematological malignancy.

	Serum VEGF levels before treatment (pg/mL) mean ± standard deviation	Serum VEGF levels after treatment (pg/mL) mean ± standard deviation	*p* value
AML	78.75 ± 33.83	100.91 ± 68	0.329
ALL	163.64 ± 95.81	86.57 ± 60.99	0.008
CML	1011.5 ± 789.09	294.84 ± 401.17	0.037
CLL	157.50 ± 17.67	75 ± 14.14	0.019
MM	229.2 ± 81.90	125.1 ± 59.56	0.002
NHL	105 ± 21.21	79.50 ± 21.92	0.012

AML, acute myeloid leukemia; ALL, acute lymphoid leukemia; CML, chronic myeloid leukemia; CLL, chronic lymphoid leukemia; MM, multiple myeloma; NHL, Non-Hodgkin's Lymphoma.
